# Evolution of the Experimental Models of Cholangiocarcinoma

**DOI:** 10.3390/cancers12082308

**Published:** 2020-08-17

**Authors:** Annamaria Massa, Chiara Varamo, Francesca Vita, Simona Tavolari, Caterina Peraldo-Neia, Giovanni Brandi, Alessandro Rizzo, Giuliana Cavalloni, Massimo Aglietta

**Affiliations:** 1 Division of Medical Oncology, Candiolo Cancer Institute, FPO-IRCCS, Candiolo, 10060 Torino, Italy; annamaria.massa@ircc.it (A.M.); giuliana.cavalloni@ircc.it (G.C.); 2 Department of Oncology, University of Turin, 10126 Torino, Italy; chiara.varamo@ircc.it (C.V.); francesca.vita@ircc.it (F.V.); 3 Department of Oncology, Laboratory of Tumor Inflammation and Angiogenesis, B3000 KU Leuven, Belgium; 4 Center for Applied Biomedical Research, S. Orsola-Malpighi University Hospital, 40138 Bologna, Italy; simona.tavolari@unibo.it; 5 Laboratory of Cancer Genomics, Fondazione Edo ed Elvo Tempia, 13900 Biella, Italy; caterina.peraldoneia@ircc.it; 6 Department of Experimental, Diagnostic and Specialty Medicine, S. Orsola-Malpighi University Hospital, 40138 Bologna, Italy; giovanni.brandi@unibo.it (G.B.); rizzo.alessandro179@gmail.com (A.R.)

**Keywords:** cholangiocarcinoma, experimental models, animal models, cell lines, tumor microenvironment, spheroids, cancer stem cells, organoids

## Abstract

Cholangiocarcinoma (CCA) is a rare, aggressive disease with poor overall survival. In advanced cases, surgery is often not possible or fails; in addition, there is a lack of effective and specific therapies. Multidisciplinary approaches and advanced technologies have improved the knowledge of CCA molecular pathogenesis, highlighting its extreme heterogeneity and high frequency of genetic and molecular aberrations. Effective preclinical models, therefore, should be based on a comparable level of complexity. In the past years, there has been a consistent increase in the number of available CCA models. The exploitation of even more complex CCA models is rising. Examples are the use of CRISPR/Cas9 or stabilized organoids for *in vitro* studies, as well as patient-derived xenografts or transgenic mouse models for *in vivo* applications. Here, we examine the available preclinical CCA models exploited to investigate: (i) carcinogenesis processes from initiation to progression; and (ii) tools for personalized therapy and innovative therapeutic approaches, including chemotherapy and immune/targeted therapies. For each model, we describe the potential applications, highlighting both its advantages and limits.

## 1. Introduction

Cholangiocarcinoma (CCA) is a heterogeneous group of tumors originating from the epithelium of biliary tract. It represents the second most common primary liver malignancy after hepatocellular carcinoma (HCC) [1]. Based on its anatomical origin, CCA is classified as intrahepatic (iCCA), which accounts for 10% of CCA [2], and extrahepatic (eCCA), which is more frequent, further subdivided in perihilar (pCCA) and distal (dCCA) [3]. According to the recent WHO classification, 5th edition, iCCA is reclassified in: (i) large duct type, which is similar to extrahepatic cholangiocarcinoma; and (ii) small duct type, which resembles hepatocellular carcinoma. The two subtypes display different molecular and genetic aberrations, histological features, and clinical outcome [4].

CCA has different rates of incidence worldwide, with more cases in Eastern countries, but with an increasing rate in Western countries in the last three decades [5]. Its incidence and risk factors (e.g., primary sclerosing cholangitis, parasitic infections, chemical carcinogens, etc.) are geographically related [3,5,6,7]. CCA is an aggressive tumor, with a 5-year survival rate of 5–15% for inoperable cases [8]. The only potentially curative treatment is surgery; however, most patients are diagnosed at an advanced stage when resection is no longer possible. For unresectable or metastatic patients, the backbone treatment is gemcitabine (GEM)±platinum derivatives [9,10,11,12]. Unfortunately, the improvement in terms of survival is modest, and the efficacy of this treatment is impaired by chemoresistance [13]. Other therapeutic strategies have been tested, but due to the genetic variability and the clonal evolution of CCA, clinical studies with targeted therapies (e.g., monoclonal antibodies and tyrosine kinase inhibitors against EGFR, VEGF, FGFR2) have shown minimal success in terms of overall survival (OS) [14]. On the basis of positive results obtained in the FIGHT-202 study [15], the U.S. FDA has recently approved the anti-FGFR2 antibody pemigatinib (Pemazyre) in CCA patients harboring FGFR2 gene fusions (9–14% of patients) previously treated with chemotherapy. This trial is ongoing.

Recent advances in high-throughput techniques, such as next-generation sequencing and other “omics” approaches, have provided an unprecedented opportunity to broaden our understanding of the molecular mechanisms driving CCA carcinogenesis, leading to an extremely large body of data that must be properly interpreted and translated into clinical practice. To address this challenge, generating CCA preclinical models has become crucial for: (i) elucidating the causes and molecular mechanisms involved in carcinogenesis, tumor progression, and metastatization; (ii) discovering both prognostic biomarkers and druggable targets; and (iii) testing the therapeutic effects of drugs and developing more efficient therapies.

The aim of this review is to describe all the preclinical models used for CCA research, focusing on their strengths and weaknesses.

## 2. Evolution of *in vitro* CCA Models

### 2.1. 2D Models: Cell Lines and Primary Cell Cultures

For several years, human or animal primary cultures and established cell lines have represented important *in vitro* models, widely used in cancer research to study the biology of cancer and to test the efficacy of anticancer drugs. Cell lines retain some genetic alterations and transcriptomic profiles detected in primary human tumors, allowing high-throughput screening studies to identify potential druggable targets, prognostic and predictive biomarkers of drug responses, and investigate mechanisms of tumorigenesis and drug resistance, exploiting the recently developed advanced genetic tools. However, some cell lines, generated from metastases or from patient-derived xenografts, display a genetic and molecular spectrum that does not completely recapitulate primary tumors [16,17,18].

The first established and characterized CCA cell line, named HChol-Y1, was obtained from iCCA patients 30 years ago by Yamaguchi et al. [19]. Subsequently, several other iCCA and eCCA cell lines, derived from primary tumors, ascites, metastases, and xenografts have been described in the literature (Table 1).

Most CCA cell lines have been characterized biologically, but only a few have been profiled molecularly (e.g., mutational status, SNPs, rearrangements, alternative splicing, methylation, mRNA or non-coding RNA expression). Genomic studies on CCA patients have highlighted some frequent genomic alterations, including mutations of TP53 and SMAD4, ARID1A, ARID2, BAP1, KRAS, PIK3CA, NRAS, and IDH1 genes [20,21,22,23,24], mutations and/or amplification of the EGFR family member genes [21], and the rearrangement of the FGFR2 receptor [25]. The work of Akita et al. [26] found that IDH1 mutation is typical in iCCA derived from small bile duct as well as the loss of BAP1; in contrast, loss of SMAD4 expression, KRAS mutations, and MDM2 amplification are mainly found in large bile duct iCCA. A very recent study has focused on the in-depth characterization of 22 CCA cell lines, including gallbladder carcinoma, by exome sequencing, copy number, and RNA-seq analyses [27]. This large panel of cell lines, which differ in their site of origin, molecular alterations, and mutational status, represents a valid tool for drug screening tests, in particular for targeted therapy (Table 1).

Molecular profiling analyses of most established models have been carried out without contemplating the eCCA subdivision and before the new iCCA guidelines (e.g., 5th WHO classification) [4], thus the eCCA (pCCA/dCCA) and iCCA subcategories (large/small duct types) are often missing.

In this context, cancer cell lines may be helpful to either investigate the response to drugs (e.g., chemotherapy and targeted therapy) or to probe the mechanisms underlying a potential resistance to therapy. In fact, some cell lines can be primarily resistant, retaining this feature from the primary tumor, or can acquire resistance with drug treatment. Several mechanisms involved in chemotherapy resistance have already been elucidated, including the high expression of drug efflux pumps, the increase in detoxification of chemotherapeutic drugs, the alteration of drug targets, and the inhibition of drug-induced apoptosis [81,82].

Concerning resistance to targeted therapy (in particular to anti-EGFR, and anti-FGFR therapies) distinct mechanisms have been proposed, including mutations in the target, reactivation of the targeted pathway, or activation of alternative pathways [83,84]. Table 1 reports some chemo/targeted therapy resistant CCA models.

Important advancements in the discovery of carcinogenesis and molecular targets have been achieved *in vitro* with the powerful CRISPR/Cas9 gene editing system, a genetic tool able to identify and execute cleavage at specific DNA sites [85]. One example of CRISPR/Cas9 application in CCA is the recent work by Yoshino et al. in which the role of ARID1A (AT-rich interactive domain-containing protein 1A) was investigated by means of gene editing. Clinically, it was demonstrated that ARID1A-negative iCCA patients had a poorer outcome compared to ARID1A-positive ones, suggesting that ARID1A may have prognostic value in iCCA [86]. To explore the role of ARID1A, the authors established ARID1A-knockout (KO) human iCCA cell lines by using the CRISPR/Cas9 system. Compared to wild type (WT), ARID1A-KO cells developed a more malignant and aggressive phenotype, as demonstrated by significantly enhanced migration, invasion, and sphere-formation ability and by concomitant high expression levels and activity of the stemness gene ALDH1A1 [86]. The CRISPR/Cas9 approach is an efficient and simple method to: (i) clarify the mechanisms of tumorigenesis, (ii) identify novel molecular targets for drug development, and (iii) potentially create engineered cells for cell-based therapies [87]. However, this technology still requires improvements in terms of efficacy and safety before its clinical translation [88].

Established cancer cell lines are the most accessible and easy models used for genome editing *in vitro* and for investigating tumor evolution. However, due to increasing passage numbers, which cause variations in both genotype and phenotype, they poorly reflect the behavior and heterogeneity of tumors *in vivo* [89]. In contrast, primary monocultures created by tumor tissue dissociation [90] retain the morphological and functional characteristics of their tissue of origin, so they are often used as a source of material for “omic” and functional studies. In addition, the culture conditions of primary cells are designed to limit cell differentiation and partially preserve the cancer stem cell (CSC) subpopulation, reflecting tumor heterogeneity.

Despite these advantages, primary cells are not yet fully representative of *in vivo* tumors, since they lack the tumor microenvironment (TME). The TME is a complex structure surrounding the tumor mass composed of a cellular part (stromal cells) and of a non-cellular part (the extracellular matrix, ECM, consisting of proteoglycans, hyaluronic acid, collagen, fibronectin, and laminin) [91]. Stromal cells, including cancer-associated fibroblasts (CAFs), mesenchymal stromal cells (MSCs), immuno-inflammatory cells, and vascular endothelial cells, create a dynamic interaction with the tumor that influences and favors its survival and progression [92]. Introducing models in which CCA cell lines or primary cells are co-cultured with stromal cells has contributed to the study of both tumor progression and drug response mechanisms by focusing on the crosstalk between the tumor and its TME. Several studies have shown the possibility of isolating neoplastic cholangiocytes (method described by Fabris et al.) [93] and stromal fibroblasts (Holt’s method) [44] in order to study the interactions between CCA and mesenchymal cells, and to investigate pathways activated by their interaction [93,94,95] in 2D co-culture systems. Among all stromal cells present in the TME, CAFs are one of the most critical and abundant components. In order to investigate the molecules activated by tumor stroma-TME interaction, Ohira et al. demonstrated that, by co-culturing fibroblasts and two different iCCA cell lines, CCA-associated fibroblasts express SDF-1, promoting the invasion and migration of tumor cells via the SDF-1/CXCR4 molecular axis [96]. *In vitro* studies indicate that SDF-1 expression induces a number of pro-tumorigenic responses, such as Bcl-2, and activates the PI3K/Akt pathway, promoting increased CCA cell invasion, prolonged survival, and epithelial-to-mesenchymal transition [97].** Another study using 2D co-culture systems showed that the contact of α-Smooth Muscle-Actin-positive (α-SMA) myofibroblastic CAFs and iCCA cells increased tumor cell proliferation and switched cells into the active stages of the cell cycle [98].

In the crosstalk between CAFs and cancer cells, exosomes play an important role [91,99]. Exosomes are membrane-wrapped extracellular vesicles containing microRNAs, DNA fragments, proteins, and other soluble factors. They are an important example of communication between cancer cells and TME by inducing either anti- or pro-tumor signaling pathways [78,93,100]. A study of co-culture system between CAFs and iCCA cells demonstrated that miR-195, carried by CAF-derived exosomes, could inhibit tumor proliferation and invasion *in vitro* [101]. Another study conducted by Chen et al. demonstrated that CCA-derived exosomes support iCCA cells to escape the attack of the immune system by preventing cytokine-induced killer (CIK) cells from producing molecules with anticancer effect, such as tumor necrosis factor (TNF)-α and perforin [102]. Hence, the *in vitro* study of exosomes-TME interactions may be important for the development of new therapeutic approaches.

#### 2.1.1. Strengths and Weaknesses of 2D Models

Although cell lines have, for decades, represented a useful tool for cancer research (long-term expansion capacity, short replication doubling time, low maintenance costs, and high reproducibility of experiments) they have many limitations, namely: (i) the success rate of establishing cell lines is low (about 10%) [66] due to an inadequate amount of tumor cells in surgical or bioptic samples where the necrotic tissue is predominant; (ii) even in optimal culture conditions, not all cancer cells are able to proliferate due to the concomitant and contaminating presence of tumoral fibroblasts; (iii) artificial and stressful culture conditions and the presence of serum do not allow the maintenance of the stem compartment, and favor the accumulation of new genomic alterations; (iv) *in vitro* stabilization often promotes the selection of homogeneous cell clones, not representative of tumor genetic heterogeneity, potentially being a bias for the translation of preclinical data in a clinical context; (v) cell lines lack the cancer stem cells (CSC) subset, which is partially maintained in primary cell cultures; (vi) cell lines proliferating as monolayer cultures lack polarization; and (vii) the 2D nature of the cultures and the absence of tumor stromal cells do not recapitulate the architecture and cell interaction of the complex TME [66,89]. Unlike *in vivo* tumors, the bi-dimensional organization permits homogeneous distribution of nutrients, cell signaling molecules, oxygen, and drugs.

Regarding the more complex primary cell cultures and different co-culture systems, they present another limitation—the short period of time to reach senescence, sometimes after only a few *in vitro* passages, impedes long-term experiments and their reproducibility. Moreover, primary cultures are often laborious and less efficient. This is particularly true in highly desmoplastic tumors such as CCA, where the overgrowth of stromal cells may significantly reduce the establishment efficiency [50].

In the last few years, more innovative *in vitro* models have been developed to improve 2D primary and cell line cultures; 3D models such as spheroids and organoids, discussed below, could be more promising models [103].

### 2.2. 3D CCA Modeling Approaches

Most cell types when placed into 2D cultures lose their differentiated phenotype. Interestingly, most of these cells maintain their physiological form and function when cultured in a 3D system [104]. This observation has led to the notion that the dimension in which cells are cultured *in vitro* (2D or 3D) is a crucial determinant of cell fate. The three-dimensional structure is a crucial characteristic of tissue and organ development; this level of organization starts during embryogenesis and continues with cell-to-cell interactions [105]. Furthermore, cells are enveloped by an ECM that affects their growth, differentiation, and homeostasis [106]. The development of three-dimensional systems has improved the study of the biochemistry and biology of tumors. A 3D architecture recapitulates the overall *in vivo* structure and composition of the tumor mass (including the stem cell niche), where the spatial arrangements reflect tumor cell–TME and –ECM interactions, also mimicking the biodistribution of nutrients, oxygen, stimuli, and drugs. However, the available methods often lack a precise control of cell external structures. Recently, the rapid development of bioengineering techniques, such as bioprinting, microfluidics, and photochemistry, has provided us with advantageous tools to reconstruct *in vitro* a 3D controllable tumor cell milieu [107]. For example, various biofunctionalized hydrogels have become the ideal candidates to acquire new insights into 3D models setup [108]. In the CCA scenario, however, these approaches are still poorly investigated. Recently, a prototype of 3D primary CCA cell culture was bioprinted using a composite hydrogel system of gelatin-alginate-Matrigel^TM^ into a pre-designed grid architecture [109].

Conventional 2D cell cultures rely on the adherence to a flat and stiff surface to provide mechanical support for the cells. However, most of these 2D methods do not provide control of cell shape, which determines biophysical cues affecting cell bioactivities *in vivo* [110]. Differently, 3D microenvironments follow the distribution of cell–ECM and cell–cell interactions that influence most cellular behaviors and determine the functions of whole organs [111]. Cells in 2D move along a planar surface by generating enough traction to overcome surface inhibition. On the contrary, movements of cells in 3D arrangements are restricted by inhibition from contact with the surface, other cells, and/or the ECM [112].

Spheroid-like structures and *in vitro* multicellular tissue constructs, known as organoids, are the most frequent and well characterized 3D models of CCA.

#### 2.2.1. Spheroids

The work by Sutherland et al. [113] paved the way to 3D cultures; they were the first to discover that lung cells grown in a floating manner would generate spheroids. Large spheroids (>500 μm in diameter) organize their structure by developing an outer zone containing proliferating cells, an intermediate zone containing a few mitosis-starved and poorly oxygenated cells, and a central zone of necrosis, a main feature of tumor bulks.

Spheroids can be generated from either primary cultures or cell lines cultured as a single- or multi-cell suspensions. Single cell suspension is generally maintained in the absence of a matrix, in low-density, attachment- and serum-free conditions to allow formation of floating spheres. Tumor cells within the spheroid closely interact with each other and such cell-cell interactions affect proliferation, survival, and response to therapy. Cell–cell adhesion is reinforced by the formation of desmosomes and dermal junctions. The close interactions between cells, coupled with the deposition of several ECM proteins (collagens, fibronectin, laminin, elastin, and tenascin), increases spheroid density, forming a physical barrier that prevents and limits the transport of drugs into the spheroid mass [114]. The absence of serum and the addition of growth factors that stimulate stem cell proliferation (EGF and FGFs, insulin, and hydrocortisone) promote the enrichment of a CSC-like population. In fact, only progenitor cells are able to generate spheroids in a starved medium [115].

CSCs are defined as initiating progenitors of cancer with self-renewal capacity, representing the tumor reservoir. They express tumor-specific stem markers, are poorly represented, quiescent, and resistant to chemotherapy [116,117,118,119]. CSCs have sufficient gene alterations to initiate a tumor, and more alterations arise during their differentiation into “mature” cancer cells.

CSC-enriched spheroids provide a 3D cell models to characterize CSC and to study their role in CCA development and in chemo-targeted therapy resistance [120]. Spheroids have been established from both CCA cancer cell lines and primary cultures. The pattern of protein expression in two eCCA cell lines (Mz-ChA-1 and SK-ChA-1) undergoing spheroid formation has been recently investigated [121]. Compared to 2D cultures, eCCA spheroids showed an increased expression of several enzymes involved in glycolysis, hypoxia signaling, the protein ubiquitination pathway, the NADH repair pathway, and the degradation of superoxide radicals [72]. In another study, spheroids were established from the iCCA cell lines SG231, HUCCT1, CCLP1, and CCA4; their molecular characterization revealed an increased expression of key genes involved in pluripotency, self-renewal, drug-resistance, and survival, as well as stem-like surface markers [122]. Indeed, CCA spheroid (cholangiosphere) models, like those from other tumors, are characterized by the presence of putative CSC and by enhanced tumorigenicity in xenograft models [71] and chemoresistance [123,124]. However, CSC markers specific of CCA have not been extensively studied; immunophenotypic analysis showed the expression of canonical CSC markers (CD133, CD24, CD44, EpCAM, Sox2, Nanog, OCT3/4, CD49f, CD11, FoxA 1/2, PDX1, Sox17, CK7/19) [123,125]. CCA spheroids have been used to investigate CSC-mediated chemoresistance or specific invasive properties. For instance, Kawamoto et al. developed spheroids from an eCCA GEM-resistant cell line (TFK-1) and from iCCA cells established from a patient with a GEM-resistant tumor. They observed that Metronidazole reduced cancer stemness features in both types of GEM-resistant CCA spheroids [72]. Cholangiosphere models were also useful for studying tumor-stroma interactions by using either direct or indirect co-culture systems. In particular, Raggi et al. demonstrated that tumor associated macrophages (TAMs) exposed to conditioned medium of CSC-enriched spheroids derived from iCCA cells, increased their recruitment and polarization [122]. Differently, Campbell at al. described a model of multicellular spheroids established by co-culturing cells derived from iCCA formed in syngeneic rat liver with varying numbers of clonal α-SMA-positive CAFs from the same tumor type within a type I collagen matrix. This model closely resembled the whole tissue samples of the parental tumor [126].

#### 2.2.2. Organoids

A more recent and promising 3D culture system to bridge the gap between 2D cultures and *in vivo* animal models is represented by organoids, which are complex 3D structures with architectures and functions similar to *in vivo* organs. They originate from stem cell progenitors cultured in the presence of a scaffold (a synthetic basement membrane) that reproduces the *in vivo* ECM [127]. The ECM guarantees the interaction between tumor cells and the TME and represents a reservoir for stimuli (cytokines, chemokines, and growth factors) and enzymes of tissue remodeling [128,129]. Synthetic matrices, such as polymer hydrogels, polyethylene glycol (PEG) macromers, or collagen are the most common *in vitro* supports used to generate organoids. Organoid formation efficiency is affected by the mechanical properties of the matrix, with values mimicking physiological organ stiffness [130]. When reaching a certain size, organoids cease to proliferate and develop a necrotic core. The process of growth arrest is thought to be linked to two phenomena: a switch from a proliferative, stem-like state to a non-proliferative one, and the loss of cell viability in the inner core of the organoid. In fact, organoid vascularization remains the major challenge in this field [131].

The main feature of organoids is to recapitulate patient tumor bulk, when cultured *in vitro* or xenografted into immune-deficient mice.

Presently, organoids obtained with colorectal, pancreatic, and lung cancer tissues are reliable platforms for the identification of new therapeutic targets and drug screening [132,133,134]. Conversely, in CCA research, there are only 10 published articles about organoids (Table 2), demonstrating that these models are still poorly explored, and their reliability should be proved.

Briefly, human CCA organoid models were successfully generated from surgical specimens [135] and core needle biopsies [144]. The genetic aberrations of the parental tumor are maintained in the organoid cultures, with about 80% of concordance in terms of mutations between organoids and primary tumors [135,144,145]. Moreover, organoids are a potential tool for drug screening, as demonstrated by Broutier [135], who screened a library of 29 compounds in iCCA organoids derived from two patients. Thus, CCA organoids are promising *in vitro* models to screen drug sensitivity [138].

#### 2.2.3. Strengths and Weaknesses of 3D Models

Spheroids are reproducible culture systems with affordable production costs. Primary cultures can generate spheroids in just a few days, while some cell lines, even when cultured in the best conditions, only form cellular aggregates. Spheroids recapitulate cell interactions, but both the original tumor stroma and vascular components are missing. The genetic and molecular characteristics are maintained, suggesting that they represent a reliable model for drug response assays.

Organoids are more costly, require a longer time for their generation (even weeks), and the stabilization success is lower than for spheroids. Although organoids do not maintain the stroma and vascular components of the *in vivo* tumor, they morphologically, genotypically, and histologically resemble the primary tumor, thus representing the *in vitro* model with the highest predictive patient-specific therapy response.

**Figure 1:**
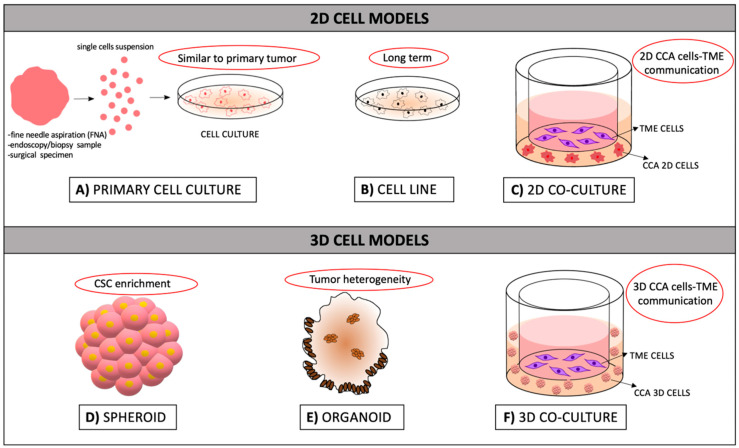
Schematic representation of *in vitro* experimental models used for investigating CCA. The upper section represents the most common 2D cell models, and the lower section shows the evolution of cell models in 3D cultures. In the red circles there are highlighted the peculiar features of each model. (**A**) Primary cell cultures were derived from surgical specimens mechanically, and enzymatically digested to obtain a single CCA cell suspension. (**B**) Established and characterized *in vitro* cell line. (**C**) Example of co-culture performed by Transwell assay to allow the crosstalk between tumor cells and TME cells; usually the TME cells are cultured in the upper compartment (e.g., stromal cells) and the CCA cells are plated in the lower compartment. (**D**) Spheroids are 3D floating cultures derived from CCA single cells, enriched in the putative CSC population. (**E**) Organoids are 3D structures with architectures and functions that are similar to *in vivo* organs; they are established from surgical specimens or biopsies, maintaining the genetic aberrations of the parental tumor. (**F**) Example of co-culture performed by Transwell assay to allow the crosstalk between 3D tumor cells, such as CCA spheroids and TME cells.

Overall, spheroids and organoids have proven to recapitulate the pathophysiological features of tumors better than 2D cell cultures, approaching the level of *in vivo* models. Undoubtedly, organoids preserve more adequately the cellular and molecular phenotypes of original patient tumors, providing a powerful tool to investigate the onset of disease, progression, as well as the development of more effective and personalized anticancer therapies. Figure 1 shows the evolution of *in vitro* cell cultures from 2D to 3D models.

## 3. Experimental Mouse Models in CCA

Animal models are an intermediate step of experimentation between 2D/3D cell cultures and human clinical trials, and represent a powerful tool to study carcinogenesis, tumor progression, and to test efficacy and toxicity of therapeutic compounds.

Compared to the *in vitro* models, animal models closely resemble physiological conditions and faithful reproduction of the tumor and its TME, allowing a thorough study of: (i) the interaction between cancer cells and the TME, and (ii) the immune and vascular system response, which cannot be investigated *in vitro*.

**Figure 2:**
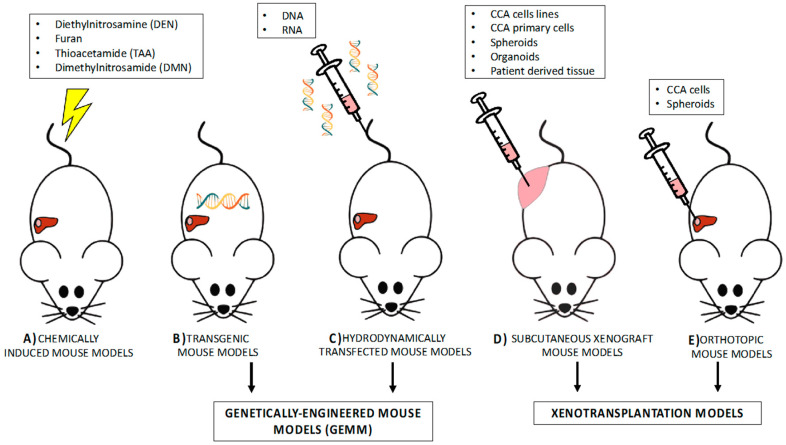
Mouse models of CCA. (**A**) Chemical carcinogens (DEN, furan, TAA, and DMN) administered to mice are able to induce CCA tumorigenesis. (**B**) Transgenic models are animals whose genome has been modified by introducing oncogenic sequences responsible for CAA induction. (**C**) Hydrodynamic transfection is used for delivering DNA or RNA into the tail vein and is considered to be an alternative for CCA germ-line knockout or CCA transgenic mice. (**B**,**C**) are included in the genetically-engineered mouse models (GEMM). (**D**) Xenografts are models of a heterotopic graft, which involve the ectopic (subcutaneous) injection of CCA human cells, spheroids, organoids, or patient-derived tissue in immunodeficient or nude mice. (**E**) Orthotopic xenograft models accurately recapitulate the TME, since CCA cells or spheroids are directly injected into mouse portal or splenic veins, or into liver parenchyma. (**D**,**E**) are of xenotransplantation models.

Of all experimental animals, mice are the most commonly and traditionally used in CCA preclinical studies; they are small, easy to manipulate, reproduce quickly, and can be genetically modified. To date, many murine models have been developed for CCA, ranging from neoplastic transformation of biliary cells to CCA progression and metastatization. Below, the most used mouse models (Figure 2) are described and subdivided, according to their application: (i) the investigation of carcinogenesis and (ii) the identification of therapeutic approaches in CCA.

### 3.1. Investigating Carcinogenesis by Means of Rodent Models

To study human carcinogenesis, animal models have been developed to reproduce human chronic inflammation associated with cholangiocarcinogenesis, and to induce or inhibit specific oncogenes or tumor-suppressor genes. Carcinogenesis models in CCA are divided into chemically induced mouse models and genetically engineered mouse models (GEMM).

#### 3.1.1. Chemically Induced Rodent Models

Chemically induced rodent models represent one of the best tools for carcinogenesis study because they mimic both tumor induction and tumor progression. Animals develop tumors by the toxic effect of specific compounds that induce DNA damage. Several studies have shown CCA formation after administering diethylnitrosamine (DEN) in mice (Figure 2A), furan and thioacetamide (TAA) mainly in rats and, dimethylnitrosamine (DMN) in hamster models [146,147].

DEN, like many nitrosamines, is a potent carcinogen able to induce liver cancer [148]. Umemura et al. demonstrated that in mice DEN (administrated in the drinking water at 20 parts per millions (p.p.m.) for 8 weeks) in combination with pentachlorophenol, an environmental pollutant (at the concentrations of 1.2, 2.2 and 2.5 mg/mouse/day for 23 weeks), promoted the formation of multifocal biliary cystic lesions and resulted in CCA development in a dose-dependent manner [149]. Proper chemically induced mouse models should better reflect the clinical background of CCA patients, such as the presence of chronic cholestasis, which has an active role in CCA onset due to the induction of genetic aberrations and pro-survival signaling pathways [146]. For this purpose, a recent model showed the tumorigenicity of intraperitoneal administration of DEN for 2 weeks prior to the left and median bile duct ligation (LMBDL), responsible for cholestasis, and DEN gavage once a week, 7 days after LDMB [150].

DMN is a potent carcinogen able to induce DNA alkylation and the production of reactive oxygen species, thus resulting in DNA damage [151]. In 1978, Thamavit et al. were the first to demonstrate the role of DMN in the induction of CCA in O. viverrini-infected hamster model. Briefly, hamsters first received an intragastric administration of O. viverrini. After the detection of the eggs, DMN was added at 0.0025% in drinking water. The authors showed that all O. viverrini-infected hamsters that received DMN developed CCA. In contrast, the group that received DMN alone and the animals only infected by O.viverrini did not develop the tumor [147].

Furan is a heterocyclic compound commonly used in animal models of liver cancer. In rats, chronic administration of 8 mg/kg furan by gavage in corn oil 5 days per week, for 15 months promoted CCA development as early as 9 months in 98% of the treated animals [152]. At higher doses (15–60 mg/kg/per day), furan administration induced rapid development of cholangiofibrosis in the caudate liver lobe after 2–3 weeks of treatment; notably, cholangiofibrosis persisted until 6 weeks after treatment, thus mimicking the natural progression from chronic bile duct lesions to cholangiofibrosis to CCA development [153].

TAA is a potent hepatotoxin able to induce progressive damage of the biliary epithelium, starting from typical dysplasia and ultimately resulting in CCA development [154]. In rats, TAA (0.03% in drinking water administrated for a 24-week period) induces hepatic fibrosis and cirrhosis; moreover it stimulates an inflammatory response in the bile ducts and an intense desmoplastic reaction, thus representing an excellent model to assess cholangiocarcinogenesis *in vivo* [155].

Chemically induced models are a useful tool to identify toxic compounds involved in CCA carcinogenesis. It should be noted that for a more detailed study, combining transgenic and chemically induced models could better clarify the mechanisms related to both tumor initiation and progression [156].

#### 3.1.2. Genetically Engineered Mouse Models (GEMM)

Genetically engineered mouse models (GEMM) harbor the most frequent oncogenic alterations (activating mutations, deletion, loss of genes) observed in human CCA. GEMMs include transgenic and hydrodynamic transfection (HT) models (Figure 2B, C). Their strength is the spontaneous formation of tumors in immunocompetent mice with an active TME, closely reflecting the human clinical condition [146].

##### 3.1.2.1. Transgenic Models

Transgenic animals are research models whose genome has been deliberately modified by introducing foreign DNA [157].

Three techniques are mainly used to create transgenic mice: (i) DNA delivery by retroviral vectors; (ii) microinjection of exogenous DNA into the pro-nuclei of fertilized one-cell embryos subsequently transferred into the oviduct of a pseudo-pregnant surrogate mother; and (iii) DNA introduction into embryonic stem cells (ES) derived from the inner cell mass of the blastocysts [157]. Currently, recombinant DNA technologies, such as the *Cre-Lox P* system, allow site- and time-specific targeting in the mouse.

The ideal transgenic model should ensure short tumor latency and the detection of metastases. In particular, CCA transgenic models should clearly show signs of inflammation or chronic liver injury before cancer development.

Several studies aimed at investigating the CCA genesis are based on transgenic mouse models that closely represent the human background of this tumor. Here, we describe their pros and cons, being conscious that the perfect CCA transgenic model does not exist yet.

Xu et al. described a model based on the conditional knockout of Smad and Pten, involved in the G1-S cell cycle arrest [158] and in cell proliferation and survival [159], respectively. Mice were affected by bile duct hyperplasia at 2 months of age, followed by iCCA development in 4–7 months. However, this model did not show any sign of chronic liver injury, inflammation, and metastases [160].

A recent report by Ikenoue et al. proposed a transgenic mouse model bearing an activating mutation of KRAS and a deletion of PTEN [161]. They demonstrated that 5-week-old transgenic mice started to develop CCA symptoms such as hemorrhagic ascites, abdominal distension secondary to hepatic enlargement, jaundice, and weight loss. The average life of mice was about 46 days and the autopsy showed several tumor nodules. Immunohistochemical analyses revealed that mice with one PTEN mutated allele had hepatocellular dysplasia with few nodules resembling iCCA, while PTEN WT mice developed only hepatocellular dysplasia, thus showing the importance of PTEN in cholangiocarcinogenesis. Unfortunately, even this model showed no inflammation or chronic liver injury before CCA development.

O′Dell et al. generated a transgenic model with two of the most common gene aberrations, involved in CCA genesis (KRAS mutation and TP53 deletion) [162]. Nine-week-old transgenic mice with homozygous deletion of TP53 already showed tumor lesions, 66% of which were exclusively iCCA and 17% had a mixed CCA-HCC phenotype. Different from the previous models, tumors showed adjacent organ invasion and metastases. Unfortunately, also in this case, transgenic mice did not show signs of inflammation or chronic liver injury [162].

The model (IDH2-KRAS) generated by Saha et al. closely represented iCCA human background, since the gain of function of both IDH1 and IDH2, involved in hepatocyte differentiation, is present in 25% of patients [24,48,163]. The authors observed palpable liver tumors in 100% mice at 33–58 weeks [48] and peritoneal metastases were detected. Subsequently, to deeply investigate the origin of the detected liver tumors, the authors performed histopathological analysis, demonstrating iCCA features by CK19 staining. However, tumor latency was long, making this model difficult to use.

Kiguchi et al. generated a model based on the constitutive expression in the liver of ERBB2 [164], often found in CCA patients. ERBB2 plays a pivotal role in proliferation and migration, as it activates the RAS-ERK and PI3K-Akt pathways [165]. They demonstrated that 85% of mice developed gallbladder adenocarcinoma at 2–3 weeks of age and that 87% and 30% of mice developed common bile duct and intrahepatic bile duct tumors, respectively, starting from 4 months of age, thus making it primarily a model of gallbladder carcinoma. Also, in this case CCA latency was too long.

The model by Zender et al. proposed a liver-specific, constitutive expression of Notch [166], a key regulator of the biliary tree proliferation in embryogenesis and involved in CCA carcinogenesis [167]. Eight-month-old mice showed alterations in the nuclear morphology of liver cells and xenotransplantation into the flanks of immunodeficient mice caused the development of iCCA, as demonstrated by desmoplastic stroma and CK7 and CK17 expression. Anyway, the authors described an HCC-CCA mixed tumor phenotype. Hence, this cannot be considered a primary CCA model.

Farazi et al. established a model based on TP53 deletion; mice were treated with carbon tetrachloride (CCl_4_), a liver fibrotic agent (10 μL/g body weight of a 10% solution in olive oil by i.p. injection three times weekly for 4 months starting at 6 weeks). Initially, mice developed chronic liver injury with fibrosis and inflammation before iCCA genesis [168], and 5% of the mice with a TP53 homozygous deletion (p53-/-) developed tumor [168]. Notably, the neoplastic lesions were embedded in a highly desmoplastic stroma, thus accurately reproducing the human disease [169]. Table 3 summarizes transgenic CCA mouse models, including both advantages and disadvantages.

Although these models reflect clinical conditions better than other models [146], they are very expensive and time-consuming. Furthermore, it should be noted that transgenes may have different expression levels than expected by means of amount and site of integration [171].

##### 3.1.2.2. Hydrodynamic Transfection for Generation of Mouse Models

Hydrodynamic transfection (HT) is a technique used for delivering DNA, RNA, proteins, and synthetic compounds to different tissues in rats, mice, dogs, and primates. Due to its high efficiency, feasibility, versatility, and safety, and its few transient side effects, HT is a promising alternative to other techniques, such as germ-line knockout or transgenic mice [172]. HT consists of a rapid tail-vein injection of a large volume (10% of body weight) [173] of the delivery solution, (such as Saline solution [174], Ringers solution [175,176], and Phosphate-Buffered Saline solution [177,178]) and, exploiting the consequent cardiac congestion and the hydrodynamic pressure in the inferior vena cava, the solution is pushed back to the liver and kidneys through the hepatic and renal vein, respectively. This pressure enlarges the pores of the fenestrated endothelium of the liver and acts on hepatocytes that are closely associated with capillaries. The membrane pores lock after injection, and the solution is entrapped inside the cells. The transfection is efficient in kidneys, spleen, lungs, and heart, but mainly in the liver, where approximately 10–40% of hepatocytes can be transfected, predominantly the cells in the peri-central region [172,179]. To overcome the rapid degradation of transfected genes, HT is used in combination with the sleeping beauty (SB) transposase that binds to specific inverted repeats and inserts said transposase at a new location in a TA dinucleotide. The HT technique is very versatile; in fact, it can be combined with other techniques, such as CRISPR-Cas9 editing [180], and it can be considered a good tool for clarifying the main players involved in CCA genesis (Figure 2C).

Among the models described in the literature, Carlson et al. [181] demonstrated that NRAS expression through HT resulted in the development of CCA nodular tumors after 4–6 weeks in Arf^−/−^ mice and after twice as long in Arf^+/−^ mice, thus confirming the already known tumor suppressive role of Arf in CCA [182]. The time required for tumorigenesis was reduced to 3–4 weeks when a constitutively activated form of AKT, implicated in cell survival and growth, was expressed in combination with NRAS, revealing also its importance in the development of iCCA [173].

Wang et al. investigated the role of Notch cascade in CCA pathogenesis by using an already established HT models of overexpressed activated forms of v-akt murine thymoma viral oncogene homolog (myr-AKT) and yes-associated protein (YapS127A) genes in hepatocytes (AKT/Yap) [183]. This previous work demonstrated that AKT/Yap-induced iCCA originate from mature hepatocyte. Unfortunately, the authors did not clarify trans-differentiation mechanisms. In this regard, Wang et al. explored the role of Notch cascade in AKT/Yap iCCA tumors. The authors confirmed that the Notch signaling pathway is activated in iCCA: in particular Notch2 is the main influencer, whereas inactivation of Notch1 slightly delays tumor development [184].

The key advantages of GEMMs consist in reproducing specific genetic aberrations identified in human tumors, and in following cancer development from its early stage. Nonetheless, in GEMM models, tumor development is slow and variable, delaying the possibility of testing therapeutic strategies. For this purpose, in order to aid the development and examination of new therapeutic responses, other mouse models, described below, are used [185].

### 3.2. Experimental Mouse Models to Investigate Therapeutic Approaches in CCA

Orthotopic and/or human tumor xenografts represent excellent models to explore cancer-related mechanisms, to identify novel therapeutic approaches, and to predict drug responses [135].

Cancer studies *in vitro* are commonly corroborated by *in vivo* tumor graft models (Figure 2D,E). Currently, it is possible to develop several kinds of grafts. Briefly, xenograft is defined as the transplantation of tissue or cells derived from a different species into an immunodeficient animal host; allograft, instead, is the transplantation of tissue or cells derived from the same species of the immunocompetent animal model [186].

#### 3.2.1. Ectopic Xenotransplantation Models of CCA

In oncology, xenografts represent a model of heterotopic graft obtained by the subcutaneous injection of human cells or tissue into the flank of immunodeficient or nude mice.

In 1985, for the first time, a human CCA cell line (SLU-132) was successfully implanted into the flank of a mouse to study the efficacy of novel anti-cancer drugs (Figure 2D) [187]. Following the success of this ectopic xenograft model of CCA, other studies have exploited cell lines derived from different CCA histotypes to investigate CCA tumorigenesis and to explore responses and safety of new therapeutic strategies. Xenograft models are advantageous because they recapitulate the genetic and epigenetic abnormalities of human tumors; moreover, they are reproducible, cost-efficient, and require short experimental times. On the other hand, a drawback of xenografts is the unfeasibility to study the crosstalk between tumors (human-derived) and the host microenvironment (murine-derived); furthermore, the site of implantation is unphysiological and rarely metastatic [188].

Ectopic xenograft models have pioneered the *in vivo* investigations focused on testing the antitumor efficacy of new therapeutic compounds (e.g., chemotherapeutic agents, targeted therapy, drug combinations) [32,54,189,190,191,192,193,194,195,196,197,198] (Figure 2D). To date, xenograft models have been obtained by the injection of 2–5 × 10^6^ suspended cells of different iCCA (e.g., HuCC-T1 and CC-LP-1) and eCCA (e.g., QBC939 and Sk-ChA-1) cell lines, with a tumor engraftment rate close to 100% and a latency of about two weeks [188]. A secondary application is the study of the role of proteins, mRNA, and non-coding RNAs on tumor biology. Recently, a novel model of a CCA xenotransplant was performed using Tet-on microRNA 21 (miR-21) organoids derived from a liver biopsy of chemoresistant iCCA. Mice were treated with a HSP90 inhibitor, or vehicle, while changes in their diet were applied to modulate the expression of miR-21. After 2 weeks of treatment, the mice were randomized to continue on doxycycline-supplemented diet or subjected to a doxycycline-free diet. The mice on doxycycline-free diet achieved a significantly better tumor response than those kept on a doxycycline diet, suggesting that miR-21, commonly overexpressed in tumors, has a role in the resistance to HSP90 inhibitors [137]. In another study, 5 × 10^6^ iCCA cells (CCLP1) transduced with a lentiviral vector carrying the pre-miR-144 were xenografted in nude mice. After 5 weeks, tumor growth was suppressed, leading to p-AKT downregulation and direct targeting of LIS1, a protein regulator involved in cancer proliferation [199]. Using a similar approach, Ursu et al. demonstrated that after 6 weeks tumor growth was significantly suppressed in mice subcutaneously injected with 1 × 10^6^ CCA cells (KMCH) stably expressing miR-876, with a mechanism involving the overexpression of the antiapoptotic gene BCL-XL [200].

In summary, these studies demonstrate the reproducibility of ectopic xenograft models, characterized by simple experimental protocols and by lack of adverse effects. Furthermore, using xenograft models, it is possible to monitor the real tumor growth by measuring the mass volume. Nevertheless, because the tumor is implanted in an ectopic site, the milieu is unphysiological and therefore not optimal to study CCA biology.

#### 3.2.2. Orthotopic Mouse Models

Orthotopic xenograft models accurately mimic the tumor milieu, favoring tumor dissemination through spontaneous metastasis. Moreover, orthotopic models allow a more realistic therapeutic outcome, fostering an adequate drug biodistribution [201]. Immunocompetent mice are used for syngeneic orthotopic implantation; they are provided with an endogenous immune system, which is crucial for immunotherapy studies in terms of the identification of therapeutic targets and predictive and prognostic factors [202]. At the same time, since there are peculiar differences between mouse and human immune systems (e.g., signaling pathways in T cells, receptor expression in immune cells, and antigen processing and presentation machinery) [203], several improvements have been introduced in mouse models to increase their heterogeneity and to better mirror human cancer milieu.

Intrahepatic engraftment is performed using two methods; the first one consists in injecting CCA cells into the portal or splenic vein of mice, and the second one consists in the direct injection into the liver parenchyma through the capsule (Figure 2E). Rizvi et al., for the first time, implanted CCA mouse cell lines into the medial lobe of mouse livers and observed orthotopic syngeneic tumor formation. The resulting tumors reflected the histopathologic characteristics typically found in human iCCA, including desmoplasia and expression of CK-19 and SRY box 9 (SOX9) [204]. Currently, this is the only CCA syngeneic mouse model available, underlying the complexity and the limitations of such models in the CCA setting. In recent studies, eCCA cell lines (EGI-1 and TFK-1 cells) expressing luciferase were transplanted into the liver through intrasplenic injection. However, these cells also engrafted into the spleen in addition to the liver [205]. Using this approach, Cadamuro et al. showed that low-dose metronomic paclitaxel treatment decreased lung dissemination of EGI-1 cells without significantly affecting their local tumor growth [206]. Erice et al. performed an orthotopic model implanting small fragments of eCCA tumor derived from EGI-1 cells previously grown subcutaneously. After monitoring tumor implantation by magnetic resonance imaging, mice were randomized to receive a diet with an agonist (Obeticholic Acid) of the bile acid receptor, which inhibited tumor growth compared to the control group [207]. Interestingly, spheroids developed from CSCs immunosorted from human CCA primary cells (approximately 1 × 10^5^ cells) and intrahepatically injected developed tumor masses only in mouse models of liver cirrhosis within 4 months; moreover, these tumors displayed epithelial traits reproducing the original human iCCA [120]. More recently, a minimally invasive ultrasound-guided intrahepatic injection was established. Due to its nominally invasive nature, this method decreases the risks of surgical complications and provides a potential experimental tool for future screening of cancer therapeutics in orthotopic models [208].

Although orthotopic implantations are an impressive tool to conduct investigations focused on the TME and tumor progression, their development requires a long period, the study of tumor spreading is laborious, and it is based on imaging tools or on animal euthanasia [146].

#### 3.2.3. Patient-Derived Xenograft (PDX)

PDX models are currently used to study tumor biology and to test personalized therapies. In this approach, tumor fragments derived from biopsies or surgical specimens are directly implanted into the dorsal region (subcutaneous implantation) or target organs (orthotopic implantation) of immunodeficient mice [209]. PDX retain the main traits of tumor bulk, including the surrounding stroma, 3D architecture, and tumor heterogeneity (Figure 2D), thus providing a powerful and reliable tool for predicting the therapeutic response in different types of solid cancers [210,211,212].

Recently, several groups successfully obtained PDX models of iCCA. The first was generated by Cavalloni et al., who established a KRAS-mutant CCA PDX model that faithfully recapitulates the histologic, genetic, immunogenetic, and transcriptomic profiles of the parental tumor [56]. This model was also used to test the *in vivo* efficacy of trabectedin and its role in deregulating RNA transcripts involved in cell adhesion, stress-related response, and in pathways involved in cholangiocarcinogenesis [213,214]. A second iCCA PDX model, endogenously expressing the FGFR2-CCDC6 fusion protein was employed to show the ability of the FGFR inhibitors ponatinib, dovitinib and BGJ398 to modulate FGFR signaling and inhibit tumor growth [215]. Moreover, Saha et al. created iCCA PDX models bearing an IDH mutation, frequently present in CCA, demonstrating a pronounced efficacy of dasatinib, a multi-tyrosine kinase inhibitor [216]. Another group demonstrated that the treatment of iCCA PDX models with a pan-FGFR inhibitor was associated with a decrease of tumor size and necrosis [217]. Garcia et al. showed that JQ1, a bromodomain and extra-domain inhibitor, suppressed tumor growth in both an iCCA and an eCCA PDX models, inhibiting c-Myc expression [218]. A study published last year showed that a selective Notch1 inhibitor switched off the Notch pathway and reduced tumor growth to the same extent of GEM in iCCA PDXs [219].

Although many CCA PDX have been established, derived from both iCCA and eCCA, only a few have been molecularly and genetically characterized. Table 4 shows some of PDX models, of which at least one molecular/genetic characteristic is known.

Unfortunately, PDX models have some crucial limitations. Firstly, the variability in the success of engraftment rate is significantly different depending on both the primary tumor itself (presence of necrotic tissue, time from surgery, and tumor engraftment and aggressiveness) and the manipulation ability in different laboratories (internal organization, different protocols for treating surgical samples, etc.). Finally, another strong limitation is the long period required for engraftment, frequently up to several months. This prolonged time should be considered as a negative factor when PDX models would be used as “Avatars” for therapy personalization.

## 4. Conclusions and Future Perspectives

Here, we have detailed the different preclinical models available for CCA studies. In the last decades, many improvements have been made; *in vitro* and *in vivo* models have achieved extensive progresses and have increased their complexity and reliability. However, all the models have advantages and drawbacks (Table 5); to date, 2D cell lines and mice xenografts, the most frequently used tools, remain the first step for all preclinical investigations, even with their well-known limits. All these models should be singularly exploited to improve our knowledge of this disease, from early onset to tailored therapies, but they are not completely representative of the human tumor. Creating a preclinical model to fully recapitulate all the tumor conditions is the new challenge.

In particular, “ideal” animal models should be developed and fully characterized, accounting for the CCA site of origin and sub-classification, their stem cell origin, and genetic, epigenetic, and molecular alterations. The various predisposing risk factors make CCA a heterogeneous malignancy. It is mandatory to develop models that recapitulate, for example, precancerous lesions due to chronic damage (e.g., chronic cholestasis, cirrhosis, primary sclerosing cholangitis, HCV and HBV infections, and steatosis), in order to investigate the disease from its origin to its progression. Furthermore, it is mandatory to standardize the CCA classification, in particular iCCA subgroups (small and large duct types), taking into account the recent WHO classification of digestive system tumors, and data relating to the differences on their genetic and molecular profiles, which reflect the diversity in terms of clinical pathological features. Failure to observe the iCCA classification could represent a limitation of current CCA preclinical models, therefore, the scientific community should adapt to the new guidelines. It is also necessary to analyze the role played by the desmoplastic stroma, characteristic of CCA, in the response to therapies. Finally, the TME, including the immune system, needs to be further explored. In recent years, immunotherapy has become increasing popular, giving encouraging results in some tumors. The creation of suitable immunocompetent models of CCA would allow to deepen the role of the immune system in the antitumor response and to investigate the applicability of immunotherapy in CCA patients to improve survival. In addition, the generation of new models of chemo/targeted therapy resistance could allow a better understanding of the mechanisms underlying drug resistance. Due to the rarity of the disease, the collaboration of the CCA scientific community is essential to accelerate this milestone.

## Figures and Tables

**Table 1:** Cholangiocarcinoma (CCA) cell lines described in the literature and some of their genetic/molecular characteristics and drug resistance.

Cell Line/References	Anatomic Site/Classification	Source	Genetic-Molecular Alterations/References	Drug Resistance/References
HChol-Y1/[19]	iCCA	PT	NA	
Oz/[28]	iCCA	Ascites/Mts	mKRAS/[27]	
HuH28 ^**a**–**c**^/[29]	iCCA	PT	mPIK3CA, mARID1A; mARID2; mMLH3; mTP53/[30]; Depmap portal	Gem ^**a**^/[31] Anti-EGFR Ab **^c^** Mek inhibitors **^c^**/[32]
CHGS/[33]	iCCA	PT	NA	
HuCC-T1 ^**c**,**d**^/[34]	iCCA	Ascites/Mts	mKRAS; iMSH6; mTP53; high level protein BAP1/ [27,35]; CCLE; Cosmic-CLP	FGFR inhibitors **^c^**/[36] EGFR inhibitors ^**d**^/[37]
RGHuCC-T1 **^b^**	iCCA	Ascites/Mts		Gem **^b^**/[38]
PCI:SG231/[39]	iCCA	PT	NA	
HuCCA-1 ^**a**^/[40]	iCCA	PT	NA	5-FU ^**a**^/[41]
KMC-1/[42]	iCCA	PDX	mKRAS/[43]	
CC-SW-1/[44]	iCCA	PT	mBRAF/Depmap portal	
CC-LP-1/[44]	iCCA	PT	high level protein BAP1; mTP53; mBAP1/[35]	
CC-LP-1GR **^b^**	iCCA	PT		Gem **^b^**/[45]
KMCH-2/[46]	iCCA-HCC	PT	NA	
ETK1/[47]	iCCA	Ascites, Mts	mPIK3C3; mTP53/Depmap portal	
RBE **^c^**/[47]	iCCA	PT	mIDH1; mBIRC6; mMSH6; mMSH3; mKRAS/ [48]; Depmap portal	FGFR inhibitors **^c^**/[36]
SSP-25 **^c^**/[47]	iCCA	PT	mTP53/Depmap portal	FGFR inhibitors **^c^**/[36]
RPMI 7451/[49]	iCCA	PT	NA	
SNU-1079 **^c^**/[50]	iCCA	PT	mIDH1; dARID1A; mPIK3AP1/[27]; Depmap portal	FGFR inhibitors **^c^**/[36]
KKU-M055/[51]	iCCA	PT	mMA2K1; high level mRNA FGFR1/[27]	
KKU-M156 ^**a**^/[51]	iCCA	PT	NA	Cis, Carbo ^**a**^/[51]
KKU-M214/[51]	iCCA	PT	NA	
KKU-M214R **^b^**	iCCA	PT		Gem, 5-FU, Doxo, PTX **^b^**/[52]
KKU-OCA17/[51]	iCCA	PT	NA	
HKGZ-CC/[52]	iCCA	PT	mKRAS; mTP53/Depmap portal	
NCC-CC1/[53]	iCCA	PDX	mKRAS; mTP53/[53]	
NCC-CC3-1/[53]	iCCA	PDX	mKRAS/[53]	
NCC-CC3-2/[53]	iCCA	PDX	mKRAS/[53]	
NCC-CC4-1/[53]	iCCA	PDX	NA	
KKU-M213/[54]	iCCA	PT	mKRAS; mTP53; mSMAD4/[27]; Depmap portal	
HCCC-9810/[55]	iCCA	PT	NA	
MT-CHC01 **^c^**/[56]	iCCA	PDX	mKRAS, aErbB2, dTP53/[56]	Anti-EGFR Ab **^c^** /[32]
MT-CHC01R1.5 **^b^**/[57]	iCCA	PDX		Gem, 5-FU, Carbo **^b^**/[57]
KKU-023/[58]	iCCA	PT	mTP53/[58]	
ZJU-1125/[59]	iCCA	PT	mTP53/[59]	
KKK-D049/[60]	iCCA	PDX	NA	
KKK-D068/[60]	iCCA	PDX	NA	
TKK/NA	iCCA	PT	high level mRNA ErbB2; aErbB2 [27]	
YSCCC NA	iCCA	PT	mTP53/Depmap portal	
YSCCCG100 **^b^**	iCCA	PT		Gem **^b^**/[61]
KKU-M139/NA	iCCA	PT	NA	
KKU-M139R **^b^**	iCCA	PT		Gem, 5-FU, Doxo, PTX **^b^**/[62]
HBDC/[63]	eCCA/pCCA	Ascites, Klatskin-Mts	NA	
SNU-1196/[50]	eCCA/pCCA	PT, Klatskin	aKRAS, mTP53; mSMAD6/[27,50]; Depmap portal	
KKU-100/[64]	eCCA/pCCA	PT, Klatskin	mKRAS; mTP53; mFGFR3/[58]; Depmap portal	
KKU-452/[58]	eCCA/pCCA	PT	mTP53/[58]	
ZJU-0826/[59]	eCCA/pCCA	PT	NA	
SNU-478/[50]	eCCA/dCCA	PT/Ampulla of Vater	mMLH1; mTP35/[50]	
SNU-869/[50]	eCCA/dCCA	PT/Ampulla of Vater	mKRAS; mPI3K, mTP53/[27,30,50]	
TGBC-51/[65]	eCCA/dCCA	PT/Ampulla of Vater	NA	
TGBC18TKB/NA	eCCA/dCCA	PT/Ampulla of Vater	mErbB2; mBRAF; high level mRNA ErbB2/[27]	
EGI-1 ^**d**/**c**^/[66]	eCCA/dCCA	PT	mKRAS; mTP53/[27]; CCLE; Cosmic-CLP	EGFR inhibitors ^**d**^/[37] Anti-EGFR Ab **^c^**/[32]
Sk-ChA-1 ^**d**^/[67]	eCCA	Ascites/Mts	mBRAF; tmRASA1/[27]	EGFR inhibitors ^**d**^/[37]
MEC/[68]	eCCA	Pleural Effusion/Mts	NA	
KMBC/[69]	eCCA	PT	NA	
TFK-1 **^c^**/[70]	eCCA/dCCA	PT	mTP53; mMSH6; mFRFR3/[71]; Depmap portal	Anti-EGFR Ab **^c^**; Mek inhibitors **^c^**/[32]
TFK-1GR **^b^**	eCCA/dCCA	PT		Gem **^b^**/[72]
OCUCh-LM1/[73]	eCCA	Liver Mts	NA	
QBC939/[74]	eCCA	PT	NA	
RGQBC39 **^b^**	eCCA	PT		Gem ^**b**^/[38]
ICBD-1/[75]	eCCA	PT	NA	
TK/[76]	eCCA	Ascites/Mts	NA	
SCK/[77]	eCCA	PT	NA	
SCK-R **^b^**	eCCA	PT		Gem; 5-FU, Cis **^b^**/[78]
JCK/[77]	eCCA	PT	NA	
Cho-CK/[77]	eCCA	PT	NA	
Choi-CK/[77]	eCCA	PT	NA	
SNU-245/[50]	eCCA/dCCA	PT	mBIRC6; mTP53/Depmap portal	
TGBC-47/[65]	eCCA	PT	NA	
TBCN-6/[65]	eCCA	PT	NA	
RMCCA-1/[79]	eCCA/pCCA	PT	NA	
NCC-BD1/[53]	eCCA	PDX	mKRAS; mTP53/[53]	
NCC-BD2/[53]	eCCA	PDX	mTP53/[53]	
KMCH-1 **^c^**/[80]	CCA/HCC	PT	mPTEN; dARID1A; mMSH4; mBRAF/OMICS; depmap portal	Anti-EGFR Ab; Mek inhibitors **^c^** /[32]

iCCA: intrahepatic cholangiocarcinoma; eCCA: extrahepatic cholangiocarcinoma; pCCA: perihilar cholangiocarcinoma; dCCA: distal cholangiocarcinoma; HCC: hepatocellular carcinoma; PT: Primary tumor; Mts: metastasis; PDX: patient-derived xenograft; NA: not available; m: mutation; tm: truncating mutation; a: amplification; d: deletion; i: insertion; CCLE Cosmic-CLP: Cancer cell line encyclopedia project; Cosmic cell line project. **^a^** cell lines with intrinsic chemotherapy resistance; **^b^** cell lines with acquired chemotherapy resistance; Gem: gemcitabine; 5-FU: 5-fluorouracil; Cis: cisplatin; Carbo: carboplatin; Doxo; doxorubicin; PTX: paclitaxel; **^c^** cell lines with intrinsic targeted therapy resistance; **^d^** cell lines with acquired targeted therapy resistance.

**Table 2:** Organoid models in CCA studies.

Organoid Source	Study Function	References
**Human CCA patient**	Drug screening Drug resistance Cancer differentiation Cell plasticity CCA metabolism Personalized Radiosensitivity	[135,136,137,138,139,140]
**Human cholangiocyte GE**	Cancer gene function	[141]
**Human Hepatocytes GE**	Cancer initiation Identification preventive therapies	[142]
**Human hepatocarcinoma patient**	Cancer differentiation Cell plasticity	[143,144]

GE: genetically engineered.

**Table 3:** CCA transgenic mouse models described in the literature.

Name	Generation	Effects	Advantages	Disadvantages	References
**Smad4-Pten model**	Smadco and Ptenco with Alb-cre mice.	Bile duct hyperplasia at 2 months; Tumor development at 4-7 months	Similar to human iCCA	Mixed HCC-CCA phenotype; No inflammation; No chronic liver injury; No metastases	[170]
**KRas-IDH model**	Mutant IDH2 (LSL-IDH2R172K), mice with activating KRAS mutation (LSL-KrasG12) with Alb-Cre mice.	Palpable liver tumors at 33-58 weeks	Peritoneal metastases; Similar to human iCCA	Long latency time	[48]
**KRas-Pten model**	Mice carrying a specific mutation of KRAS (LSL-KrasG12D) and/or a Ptenflox with Alb-Cre+ mice.	Multiple tumor nodules	Similar to human iCCA; Short tumor latency	No chronic liver injury; No metastases. No inflammation	[161]
**KRas-P53 model**	Alb-Cre mutants with KrasG12D mice with or without deletion of tumor protein 53.	Tumors develop at 9 weeks of age	Adjacent organ invasion; Distant metastases	No chronic liver injury; No stromal No inflammation;	[162]
**ErbB-2A model**	Bovine Keratin 5 (BK5) promoter in mice with constitutive expression of ErbB2.	Gallbladder carcinoma at 2-3 weeks;Bile duct carcinoma and iCCA at 4 months	Similar to human iCCA	Gallbladder carcinoma model; Long latency time	[164]
**Notch1 model**	Mice with overexpression of intracellular domain of Notch 1 (NICD) (Rosa26Notch1C) with Alb-Cre mice.	Changes in nuclear morphology at 8 months.	iCCA after xenotransplantation of malignant cells.	Probably mixed HCC-CCA phenotype due to the high plasticity of the transformed cells.	[166]
**P53-/- CCL4 model**	CCL4 in p53 deleted mice	Fibrosis with cholangiocyte proliferation; iCCA development in 54% of mice.	Chronic liver injury with fibrosis and inflammation.	Development of HCC; Long treatment with CCL4	[170]

**Table 4:** Representative and characterized CCA patient-derived xenograft (PDX) models.

Tumor Type	Mice	Molecular Characterization	References
iCCA	NOD/SCID	K-Ras mutation	[56]
iCCA	NSG	FGFR2-CCDC6 gene fusion	[215]
iCCA	NGS	DH1 R132C mutation	[216]
iCCA	NOD/SCID	Constitutive expression of FGFR 1–4 mRNA and FRS2	[217]
iCCA	CD1 immunodeficient nude	NOTCH1 overexpression	[219]
iCCA	B-NDG miceBALB/c (nu/nu) nude	CDK7 overexpression	[220]
iCCA	NOD/SCID	YAP overexpression	[204]
iCCA	Balb/c RJ mice	Oct-3/4 or Sox2 expression	[60]
iCCA	NOD/SCID	Sodium-dependent vitamin C transporter 2 (SVCT-2) expression	[221]
eCCA	Balb/c RJ mice	Oct-3/4 or Sox2 expression	[60]

iCCA: intrahepatic cholangiocarcinoma; eCCA: extrahepatic cholangiocarcinoma; NOD/SCID: nonobese diabetic/severe combined immunodeficiency; NSG: NOD-*scid IL2rγ^null^; B-NDG;* NOD-*Prkdc^scid^IL2rg^tm1^*/Bcgen.

**Table 5:** Advantages and disadvantages of *in vitro* and *in vivo* CCA experimental models.

Experimental Model			Advantages	Disadvantages
***In vitro*** **models**	**2 D models:**	**Cell lines**	Long-term expansion capacity; High reproducibility of experiments; Short replication doubling time; Low maintenance costs.	Low success rate of establishing cell lines; No preservation of the cancer stem compartment; No representative of tumor genetic heterogeneity; Lack of the TME components.
		**Primary cells**	Reflection of tumor heterogeneity; Preservation of the cancer stem compartment.	Short period of time to reach senescence; Low reproducibility of experiments; Lack of the TME components; Laborious to obtain.
	**3 D models:**	**Spheroids**	Recapitulation of cell interactions; Maintenance of cell physiological form and function; Reliable model for drug response assays; Enrichment of cancer stem cells population; Low production costs.	Difficult long-term maintenance; Lack TME components; Simplified architecture; Uncontrollable size and composition.
		**Organoids**	Resemble the primary tumor morphologically, genotypically and histologically; Maintenance of cell physiological form and function. *In vitro* model with the highest predictive patient-specific therapy response.	Low stabilization success; High production costs; Lack of tumor stroma and vascular components.
**Experimental Model**			**Advantages**	**Disadvantages**
**In vivo models**	**Chemically induced models**		Mimic both tumor induction and tumor progression; Identification of carcinogenic compounds; Cheap and easy to obtain.	Possible toxicity on other organs; No chance to study the role of a specific genetic mutation.
	**Genetically engineered models**		Close to human clinical condition; Spontaneous development of tumors in immunocompetent mice with an active TME; Reproduction of specific genetic aberrations of human tumors; Possibility to study cancer from its early stage.	Expensive and time-consuming; Transgenic models may have different qualitative and quantitative expression levels of integration; Tumor development is slow and variable, delay in testing therapeutic strategies.
	**Xenograft models:**	**Ectopic model**	Test of therapeutic drugs; Simple protocol and easily reproducible; Lack of adverse effects in the animal; Monitor the real tumor growth by measuring the tumor mass volume.	Severe discrepancies between the origin of the tumor and the host tissue; Unphysiological implantation, rarely metastatic;Lack of immune system.
		**Orthotopic model**	Mimic the tumor milieu; Spontaneous metastases; Test of therapeutic drugs.	Time-consuming; Tumor monitoring is laborious and based on imaging tools or on animal euthanasia.
		**Patient Derived Xenograft**	Study for personalized therapy; Retention of the stroma, 3D architecture and heterogeneity of tumor bulk.	Time-consuming; Variability in the success of engraftment.
		**Syngeneic model**	Use of immunocompetent animals; Ideal for immunotherapeutic assessment; Test of therapeutic drugs.	Time-consuming; Biology and tumor stroma of animals, far from the human condition; Not proper for human personalized medicine.
